# Using a theory informed approach to design, execute, and evaluate implementation strategies to support offering reproductive genetic carrier screening in Australia

**DOI:** 10.1186/s12913-023-10053-1

**Published:** 2023-11-20

**Authors:** Stephanie Best, Janet C. Long, Zoe Fehlberg, Natalie Taylor, Louise A. Ellis, Kirsten Boggs, Jeffrey Braithwaite

**Affiliations:** 1 Australian Institute of Heath Innovation, Macquarie University, Sydney, Australia; 2 Australian Genomics, Murdoch Children’s Research Institute, Melbourne, Australia; 3 Department of Health Services Research, Peter MacCallum Cancer Centre, Melbourne, Australia; 4 Victorian Comprehensive Cancer Centre Alliance, Melbourne, Australia; 5 Sir Peter MacCallum Department of Oncology, University of Melbourne, Melbourne, Australia; 6 School of Population Health, Faculty of Medicine and Health, University of New South Wales, Sydney, Australia; 7 Department of Clinical Genetics, Sydney Children’s Hospitals Network-Westmead, Sydney, Australia; 8 Centre for Clinical Genetics, Sydney Children’s Hospitals Network-Randwick, Sydney, Australia

**Keywords:** Reproductive genetic carrier screening, Implementation strategy, Health care professionals, Theoretical domains framework, Primary care

## Abstract

**Background:**

Health care professionals play a central role in offering reproductive genetic carrier screening but face challenges when integrating the offer into practice. The aim of this study was to design, execute, and evaluate theory-informed implementation strategies to support health care professionals in offering carrier screening.

**Methods:**

An exploratory multi-method approach was systematically employed based on the Theoretical Domain Framework (TDF). Implementation strategies were designed by aligning TDF barriers reported by health care professionals involved in a large carrier screening study, to behaviour change techniques combined with study genetic counsellors’ experiential knowledge. The strategies were trialled with a subset of health care professionals and evaluated against controls, using findings from questionnaires and interviews with healthcare professionals. The primary outcome measure was the number of couples who initiated enrolment.

**Results:**

Health care professionals (*n* = 151) reported barriers in the TDF Domains of *skills,* e.g., lack of practice in offering screening, and challenges of *environmental context and resources,* e.g., lack of time, which informed the design of a skills video and a waiting room poster using the TDF-behaviour change technique linking tool. Following implementation, (Skills video *n* = 29 vs control *n* = 31 and Poster *n* = 46 vs control *n* = 34) TDF barrier scores decreased across all groups and little change was observed in the primary outcome measure. The skills video, though welcomed by health care professionals, was reportedly too long at seven minutes. The waiting room poster was seen as easily implementable.

**Conclusions:**

As carrier screening moves towards mainstream healthcare, health care professionals report barriers to offering screening. To meet their needs, developing and testing experiential and theory-informed strategies that acknowledge contextual factors are essential.

**Supplementary Information:**

The online version contains supplementary material available at 10.1186/s12913-023-10053-1.

## Background

Interest in reproductive genetic carrier screening is building as awareness grows of the genetic risk for couples with no family history, many of whom will unknowingly be carriers of genetic conditions [1]. Carrier screening offers the potential of informing prospective parents of their chance of passing on a genetic condition to their child. Condition specific screening, e.g., for Tay-Sachs disease and cystic fibrosis, has been available for some time [2]; however, with the advent of genomic technologies, the potential to use one test to screen for a wider array of conditions is now possible and increasingly affordable [3, 4]. Advising couples of their reproductive risk is information most couples welcome [1] and allows them to make informed decisions about alternative reproductive options, e.g., in-vitro fertilisations with pre-implantation genetic testing or prenatal testing, should they wish to access them.

Internationally, professional bodies are increasingly making recommendations that health care professionals (HCPs) providing antenatal care such as obstetricians and fertility specialists (e.g., in Australia, the Netherlands and the USA) should offer carrier screening to their patients with the aim of ensuring people can make an informed decision about screening in line with their values [5–7]. However, uptake amongst HCPs varies, with professionals such as obstetricians more readily incorporating discussion around carrier screening into their practice [8]. Other HCPs such as General Practitioners (GPs) also play a key role in providing information and facilitating decision making during the pre-pregnancy and antenatal period [9]. However, GPs perceive a range of barriers to engaging with carrier screening including low confidence in their knowledge and skills, varying interest in the area, concern over routinising testing/medicalising pregnancy, and time constraints during appointments, amongst others [9]. This multi-layered picture demands a nuanced approach to designing, executing, and evaluating implementation strategies to support HCPs to offer carrier screening where consideration is given to the interplay of context, content, and implementation [10]. An understanding of context requires involvement from those working in the field [11].

As screening has become more complex, genetic counsellors’ (GCs’) practice has evolved to include supporting not only couples and individuals undertaking screening but also in the establishment of carrier screening programmes and the clinicians offering screening [12, 13]. GCs are specialised Allied Health Professionals who work with individuals, couples and families to help them understand and adapt to their risk of genetic disease. In carrier screening, a GC’s primary role is to support couples who return an increased chance result. This includes giving information about the condition and inheritance pattern as well as talking them through their reproductive options. Secondary to this, GCs work with primary healthcare providers such as GPs to support them in offering carrier screening to couples. This includes providing education about carrier screening to HCPs as well as being available to answer questions they or their patients may have. The study GCs for Mackenzie’s Mission played a key role in providing HCP education and support in addition to providing counselling for people identified to be at increased chance result.

In clinical practice, strategies to support the implementation of an evidence-based practice are commonly informed by clinician intuition, making replication and generalisation challenging [14]. The use of theory can address this hurdle, allowing learning, and leading to replicable implementation strategies. To design implementation strategies, it is essential to complement contextual knowledge with theory [15]. For example, the Theoretical Domains Framework (TDF), a psychosocial behavioural framework synthesised from 33 behaviour and behaviour change theories [16], can be used to understand influences on behaviours and was used in this study. The TDF provides a wide ranging in-depth behavioural theory-informed framework through which to investigate a topic and provide an avenue to associated implementation strategies Domains include, for example, skills (i.e., an ability or proficiency acquired through practice), professional identity (i.e., a coherent set of behaviours and displayed personal qualities of an individual in a social or work setting) and emotion (i.e., a complex reaction pattern, involving experiential, behavioural, and physiological elements, by which the individual attempts to deal with a personally significant matter or event) [17]. Identifying which domains present as barriers to clinicians’ target behaviour (here, offering carrier screening) permits a theory-informed approach to generating implementation strategies to address them.

### Reproductive genetic carrier screening in Australia and study context

**Fig. 1:**
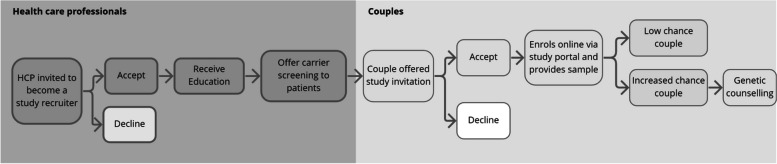
Pathway to Participation in Mackenzie’s Mission for Health Care Professionals (HCPs) and Couples

Australia has a public healthcare system, however, many tests such as carrier screening are predominantly accessed on a user-pays basis. This study was conducted within Mackenzie’s Mission, a national research programme investigating the provision of carrier screening for around 750 conditions free of charge to thousands of reproductive couples across Australia. The programme details are reported elsewhere [18], in summary, Mackenzie’s Mission was designed to recruit couples via their HCPs, mirroring other pre- and early pregnancy screening (i.e., non-invasive prenatal screening and maternal serum screening). HCPs from a range of settings across Australia including those serving regional, rural and remote areas, Indigenous communities and areas of high to low socio-economic status, were invited to participate via the study GCs. HCPs were identified through multiple routes including via existing networks, Primary Healthcare Networks (groups of independent Australian Government-funded organizations that coordinate primary healthcare in their region); practitioner education events; online searches of practitioners in specific geographic areas e.g., rural and remote; posts on relevant social media groups; presentations at conferences; professional networks; word of mouth; and snowball sampling [18]. If they accepted, HCPs were provided with education by the study GC about carrier screening and offering the test to their patients through the study. HCP education includes information about the Mackenzie’s Mission study and information on carrier screening and how to offer screening to their patients [18]. During routine appointments, HCPs could discuss carrier screening with patients who were planning or were in early pregnancy and directed the couple to a study website. Following the offer from the HCP, the couple could then log onto the study portal, complete consumer education, and at this point decide if they wished to accept or decline carrier screening. A cheek-swab would then be sent to the couple from a participating laboratory. Test results were returned online, or if found to be carriers giving them an ‘increased chance’ (usually 1 in 4 of having a child with a genetic condition), a study GC in discussion with the HCP, would arrange post-test counselling. Figure 1 details HCP and couples’ pathway to participation in Mackenzie’s Mission.

The aim of this study was to design, execute and evaluate implementation strategies to support HCPs offering carrier screening.

## Methods

### Research design

To investigate possible implementation strategies that may support HCPs to offering carrier screening to their patients, we used an exploratory multi-method approach with a pre and post cohort study design. This design allowed the collation and triangulation of a range of data, including questionnaires, interviews informed by direct observations and field notes. This study was structured using French et al.’s systematic step wise approach based on the TDF [19].

### Participants and recruitment

This study involved two cohorts. HPCs, in particular GPs, but also obstetricians and genetics health professionals interested in becoming recruiters for Mackenzie’s Mission were eligible to complete a questionnaire prior to education to identify the perceived barriers to offering carrier screening. Second, once the barriers were known and strategies designed, a subset of recruiting GPs were selected by the study GCs and, using a non-randomised method, were allocated to one implementation strategy or as a control as a comparison matched on size, geographic location and socio-economic status of the HCP clinic area and populations.

We also captured data from the study GCs. This group, located across Australia, were experienced in discussing genetic health with patients and highly skilled in providing counselling to align patients’ values and decision making.

### Procedure

Three distinct phases of research were undertaken to investigate theory-informed implementation strategies (Table 1). Throughout the project the implementation science study team regularly attended operational and research meetings providing an opportunity for direct observations of the progress of the study and keeping field notes to inform the three phases. These observations provided context for the data analysis, in particular the study GC debriefs and meetings.

**Table 1:** Three phases to investigate theory-informed implementation strategies

Phase	Data collection	What was involved		
Phase 1: Design of the implementation strategies to support HCPs offer carrier screening	HCP Pre questionnaire Study GC discussions and workshop	Identify TDF barriers to offering carrier screening	Link TDF barriers with BCTs to generate evidence and theory-informed strategies	Sense check strategies with study GCs
Phase 2: Execution of the strategies in practice	Study GC debriefs (fortnightly) Study GC meetings (monthly)	Identify clinics to receive one strategy and comparison clinics	Implement strategies	Collect evaluation data
Phase 3: Evaluation of the strategies	HCP Post questionnaire	Analyse evaluation data		

*Key*: *TDF* Theoretical Domains Framework, *HCP* Health Care Professional, *GC* Genetic Counsellor, *BCT* Behaviour Change Techniques

#### Phase 1 Designing implementation strategies

To facilitate the design of evidence-informed strategies, HCPs were invited to complete a validated TDF-informed questionnaire [20] which we examined to assess the internal reliability of the questionnaire in this context. The questionnaire was administered online or in hardcopy prior to the Mackenzie’s Mission study education session and included 35 statements about the target behaviour “offering carrier screening”. Participants were asked to indicate their level of agreement to the statements on a five-point Likert scale ranging from 1 “strongly agree” to 5 “strongly disagree”. Participants were also asked to rank their familiarity with the relevant practice guidelines on a comparable scale. During analysis, the statements were categorised to the TDF domains and can be used to identify the TDF domains that are perceived as barriers (Table 2). Following analysis, the identified TDF barriers were cross-referenced against the online interactive Theory and Techniques Tool; https://theoryandtechniquetool.humanbehaviourchange.org/tool [21]. The tool links TDF domains with evidence and theory-informed behaviour change techniques (BCTs) (e.g., Knowledge ‘*the awareness of the existence of something*’ links with ‘*instruction to perform a behaviour*’). Prior to implementation we shared a list of potential strategies that were flagged as being evidence-based with the study GCs through a series of interviews and a workshop held May 2020. This step ensured the strategies were contextually appropriate and potentially implementable (Supplementary Material 1). The final strategies designed are reported in the results.

**Table 2:** TDF domains mapped to questionnaire statements

Statements (ended with target behaviour… ‘*offer* carrier screening’)	TDF Domain
I know what the guidelines say about the need to … I fully agree with the guidelines which instruct staff to …	Knowledge
^a^Training has not been offered to me to … ^a^Training is not adequate to …	Skills
^a^It isn’t my responsibility to … I am clear about what my role should be in the process to …	Social/professional role & identity
^a^I do not find it easy to … ^a^I have previously encountered problems on similar referrals when trying to …	Beliefs about capabilities
^a^It does not matter too much if I do not… It will be bad for the patient if I do not …	Beliefs about consequences
^a^Emergencies and other priorities get in the way of me being able to … ^a^Other guidelines conflict with trying to …	Motivation & goals
I habitually (or usually) … ^a^There are justifiable reasons for why I would decide not to …	Memory, attention & decision processes
^a^There is not a good enough system in place to … I have the necessary resources (e.g., correct/enough equipment, staff, etc.) to … Verbal and written communication between staff is clear enough for me to …	Environmental context & resources
^a^My colleagues don’t seem to … My professional body would like me to …	Social influences
^a^I feel anxious if I think about having to … ^a^I worry if I think about having to …	Emotion
^a^Plans in my head often get muddled when trying to … ^a^Things are too unpredictable to make plans to …	Behavioural regulation & action planning
^a^There are more important things to achieve than making sure I… I have a system that helps me plan to … ^a^It conflicts directly with other things I am trying to achieve if I …	Goals
I am committed to … I am open to changing aspects of my work/practice in order to … I will follow recommendations (e.g. RANZCOG/RACGP) that will help to …	Intentions
^a^There are no incentives for me to … There are intrinsic rewards (e.g., feeling good) if I … There are external rewards (e.g. saving time, resources) if I …	Reinforcement
Patient outcomes will be better if I … Patient outcomes will be worse if I … It will make a worthwhile difference to patients if I …	Optimism

*Key*: *RANZCOG* Royal Australian and New Zealand College of Obstetricians and Gynaecologists, *RACGP* Royal Australian College of General Practitioners

^a^Statements are negatively worded and reverse scored for analysis

#### Phase 2 Execution of the strategies in practice

Once implementation strategies were developed, a non-randomised method was used by the study GCs to allocate a subgroup of GP clinics to receive an implementation strategy or as the comparison. The strategies were primarily designed to be easily administered, e.g., incorporated in the education session, or included in the study welcome pack. Study GCs were not required to follow-up on the strategies. Throughout the execution phase, the research team held structured study GC meetings and met both fortnightly with the individual study GCs, (rather than burdening them to keep research journals) and monthly as a group. Fortnightly debriefs were guided by a series of questions (Supplementary Material 2) designed to monitor the strategy, e.g. “Have there been any comments from HCPs about the strategy?” Monthly meetings were held to facilitate group discussion around preliminary observations and findings. Meeting notes were taken and recorded.

#### Phase 3 Evaluation of the strategies

We evaluated the effect of the strategies on the target behaviour using the previously described TDF informed questionnaire, (Table 2) repeated pre- and 8 weeks post- implementation. HCPs did not record how many times they ‘offered carrier screening’ and so, the number of couples who initiated enrolment (i.e., a couple logging into the study portal with a unique code supplied by the HCP) in the study over the 8-week period was analysed as a proxy. Couples were *not* required to complete enrolment and consent to screening through the study. Enrolment data was collected as part of the Mackenzie’s Mission study and stored in the research management software REDCap. Data from structured study GC meetings were used to inform the evaluation of the strategies. In addition, 31 semi-structured interviews were conducted with HCPs using purposive sampling to achieve maximum variation in geographic location and experience of carrier screening. HCP interviews were framed using the TDF to gather detail on barriers and enablers to offering carrier screening. The aim of these interviews was to capture in-depth HCP experiences. Further detailed reporting on these interviews can be found elsewhere [22].

### Data analysis

#### TDF questionnaire

Descriptive analyses were used to describe participant characteristics, TDF questionnaire data and study enrolment data. The Spearman-Brown prediction formula was used to test for internal reliability for questionnaire domains with two items (with > 0.50 considered acceptable); [23–25] and Cronbach’s alpha for three items domains (with a > 0.70 being considered acceptable); [26]. Negatively worded items were reverse scored, resulting in higher scores representing a more significant barrier. Analysis was completed using SPSS Statistics version 27 and STATA SE version 17.

Internal reliability was examined for two-item TDF domains using Spearman-Brown and three-item domains using Cronbach’s alpha, with adequate reliability coefficients (0.501—0.862) demonstrated for nine of the 15 domains. TDF domains that demonstrated adequate internal reliability and were ‘Knowledge’, ‘Skills’, ‘Beliefs about consequences’, ‘Motivation and goals’, ‘Environmental context and resources’, ‘Emotion’, ‘Behavioural regulation & action planning’ ‘Intentions’, and ‘Optimism’ and were included in further analysis (Supplementary Material 3). Mean scores for each of the TDF domains were computed for each professional group. Higher means indicate stronger agreement that the items were barriers.

#### Structured Study GC meetings

Data collected from the structured study GC meetings were analysed (SB, ZF) to augment the findings from execution of the implementation strategies. Comments related to implementation such as barriers, enablers and context were noted for each approach.

#### Study GC workshop and monthly meetings

These sessions were not audio recorded to promote open and frank discussions. The meeting notes were used to identify influences on the implementation including barriers, enablers, and context for each implementation strategy.

#### HCP interviews

HCP interviews were audio recorded and transcribed. Inductive content analysis [27], was undertaken, following familiarisation with the data, with independent analysis (by SB, ZF) of five transcripts. One researcher (ZF) completed the coding with regular meetings (by SB, JCL) to refine and address any challenging coding. Recurring themes discussed by HCPs about the implementation or impact of the strategy they received were identified.

## Results

### Phase 1: Design of the implementation strategies

**Fig. 2:**
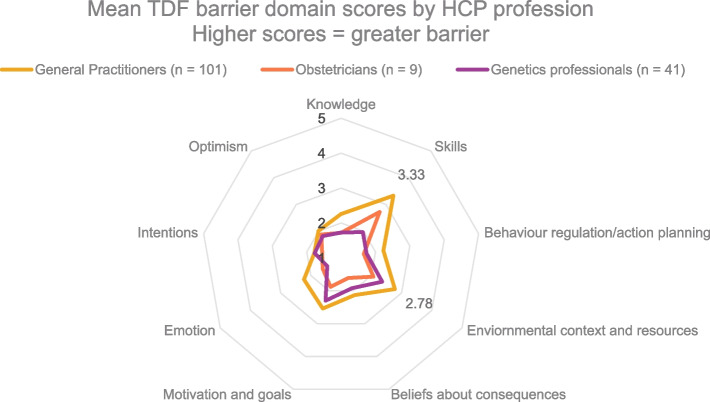
Mean TDF (Theoretical Domain Framework) barrier domain scores by HCP (health care practitioner) role

In total, 151 questionnaires were completed and used to inform the design of implementation strategies. Two thirds of participants were GPs (*n* = 101). The remaining participants were clinical geneticists or clinical GCs (*n* = 41) and obstetricians (*n* = 9). Results showed that GPs perceived more barriers than other health professionals (Fig. 2). The most common barrier domain for GPs were ‘Skills’ (*M* = 3.33, *SD* = 0.87) e.g., lack of practice in offering screening, followed by ‘Environmental context and resources’ (*M* = 2.78, *SD* = 0.79) e.g., lack of time [22]. GPs were also more likely to lack confidence in reciting the guidelines (*M* = 3.06, *SD* = 1.25) compared with genetic professionals (*M* = 2.25, *SD* = 1.17) and obstetricians (*M* = 1.33, *SD* = 0.50).

Using the Theory and Techniques Tool, theory-informed relevant BCTs were linked to the highest scoring TDF domains of ‘Skills’, including BCT ‘*instructions on how to perform behaviour*’, and for ‘Environmental context and resources’ the BCT of ‘*prompts/cues*’ (Table 3).

**Table 3:**
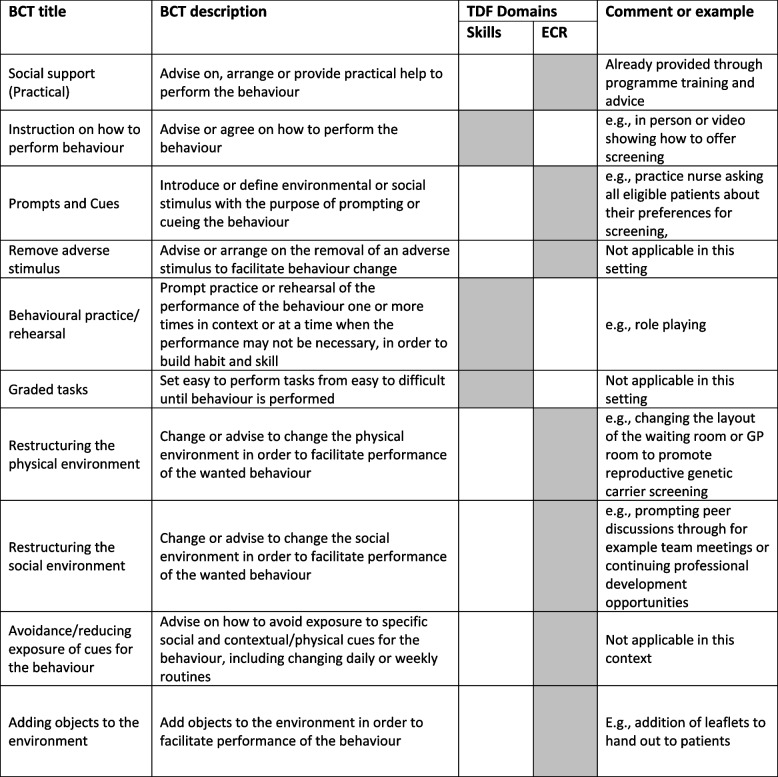
Behaviour Change Techniques (BCT) linked with identified TDF Domains shaded in grey, with examples

Skills—An awareness of the existence of something; Environmental Context and Resources (ECR)—Any circumstance of a person's situation or environment that discourages or encourages the development of skills and abilities, independence, social competence, and adaptive behaviour

*Key*: *BCT* Behaviour Change Technique, *TDF* Theoretical Domains Framework, *ECR* Environmental Context and Resources, *GP* General Practitioner, Shaded boxes indicate theoretical alignment of TDF domain with BCT

The study GCs were not trained in the use of the TDF, BCTs and the design of implementation strategies. As such the implementation science team shared potential the BCTs with the study GCs reference group who had reported a range of implementation strategies. We held a workshop to discuss aligning potential implementation strategies with theory whilst striving for a balance between supporting HCPs and while not taking up too much of their time e.g., providing feedback to HCPs to upskill them, and newsletters. Suggested strategies were refined with the study GCs through the workshop (see Supplementary Material 1) and the final implementation strategies deployed were a skills video and a waiting room poster (see Table 4).

**Table 4:** Selecting implementation strategies

Highest reported TDF domains from questionnaire	Matched BCT	Selected implementation strategy	Description of implementation strategy
Skills	Instruction on how to perform behaviour	Skills video	The video depicted a role-play of a GP offering carrier screening opportunistically to a patient during a cervical cancer screening appointment. The video ran for around seven minutes and was shown as part of the education session run by a study GC prior to HCPs being able to invite their patients to participate in Mackenzie’s Mission.
Environmental context and resources	Prompts/cues	Waiting room poster	A4 waiting room posters which prompt patients ‘*Thinking about pregnancy? Ask your doctor about important tests to consider before becoming pregnant’*. Posters were included in the study welcome pack sent via the post to HCPs.

*Key*: *GP* General Practitioner, *HCP* Health Care Professional, *GC* Genetic Counsellor, *BCT* Behaviour Change Techniques, *TDF* Theoretical Domains Framework

### Phase 2 Execution of the implementation strategies

Table 5 details the number of participants, clinics, and clinic location by implementation strategy.

**Table 5:** Participant characteristics by implementation strategy

	Skills video	No skills video	Poster	No poster
Number of clinics	12	14	22	22
Number of GPs	29	31	46	34
Clinic location				
Metropolitan	19	25	16	16
Regional	9	6	6	5
Remote	0	0	0	1

*Key*: *GP* General Practitioner

#### Skills video

Twenty-nine GPs from 12 clinics received the skills video and 31 GPs from 14 clinics were selected as the comparison group. In the structured meetings, study GCs reported that technical issues prevented them being able to show the skills video during education sessions. Instead, the video was sent to GPs following education, which meant it was not possible to know if GPs had watched the video. Indeed, when asked about the skills video in an interview, one GP stated ‘*I remember some technical issues. I have a feeling it was possible to view it elsewhere, but I can’t remember if I did or not. I may have looked at the video, I think I might have*.’ (GP-12, metro). Due to the length of the video (seven minutes), study GCs reported it was best shown to large groups and when education was delivered in person by a GC rather than small one-on-one sessions. Study GCs also reported the video was better received when GPs were unsure how to approach offering carrier screening. Upon reflection, the study GCs felt the addition of the skills video resulted in the inclusion of too many videos in the education session and did not suit the ambition to make the sessions succinct and conversational. Rather, study GCs considered the skills video would be an appropriate additional resource for HCPs to access in their own time should they wish.

#### Waiting room poster

Forty-six GPs across 22 clinics were provided posters and 34 GPs across 21 clinics were selected as a comparison group. One challenge with implementing the posters, reported in the structured study GC meetings, was that study GCs were unsure whether the clinics had them displayed. Study GCs also reported that clinics were busier than normal due to the COVID-19 pandemic which may have affected the use of the posters. HCP interviews also indicated ‘*it was a difficult time for any intervention. We had stripped all of our brochures, all of our magazines, anything people could touch … all the toys were gone. We didn’t take down all the posters off the walls, but we did have COVID posters*’ (GP-25, metro). Further, a greater number of appointments were conducted via Telehealth, reducing the access for patients to view physical posters in waiting rooms. As a solution, some GPs mentioned that their practice website would be a suitable place to have a digital poster though this was not trialled.

### Phase 3: Evaluating the implementation strategies

#### Skills video

Overall, the skills video elicited a mixed response from GPs but improved perceived barriers. GPs who received the skills video had less initiated enrolments in their first eight weeks (*M* = 0.79, *SD* = 1.08, 0–3 vs *M* = 1.09, *SD* = 2.30, 0–12). Pre- and post- implementation mean TDF scores for both groups (see Supplementary Material 4) showed that GPs who received the skills video reported a greater improvement in how they perceived their skills (skills video -0.90 vs no skills video -0.41). Whilst study GCs reported a positive response to the videos, some GPs did not find the videos appealing and felt they already had the skills required to offer carrier screening. As one interviewee said, ‘*I guess I didn’t find that a particular barrier, I guess because I’m already quite used to talking about offering that screening*’ (GP-05, Metro). However, one GP who lacked experience explained in an interview how ‘*I saw the video of the GP explaining it, the training bit and that looked fairly straightforward*’ (GP-21, Regional).

#### Waiting room posters

Overall, the posters were welcomed by GPs but showed limited effect on GPs behaviours. GPs who received the poster had fewer couples initiated enrolment in their first eight weeks of recruiting (*M* = 1.47, *SD* = 2.62, 0–15 vs *M* = 1.70, *SD* = 2.30, 0–9). Pre- and post-implementation questionnaire responses showed both groups (see Supplementary Material 4) perceived ‘*Environmental context and resources*’ as less of a barrier post-implementation (posters -0.56 and non-posters -0.20).

When asked in interviews whether a poster or pamphlet might be useful in raising carrier screening awareness and increasing consumer driven enquiry, HCPs were supportive of the idea ‘… *having something in the waiting room I think would also be good, because often the doctors aren’t thinking about it, it’s not at the front of their minds, and you might even have the mother of a couple who looks at the poster and thinks, well my daughter’s thinking about that*’ (GP-12, metro). GPs mentioned that displaying the poster in the clinic room helped remind them to mention carrier screening to appropriate patients. GPs spoke positively to the study GCs when asked about posters, and one GP who was in the comparison group asked if there was poster they could use. However, one participant felt ‘*People connect with stories*’ (GP-19, Metro) and therefore a picture-based approach, communicating the potential benefits of carrier screening could have had a greater impact on patients. Another mentioned the poster could have been bigger than A4 and others noted that the posters were ‘*probably being lost by all of the face masks, you know, don’t enter, check-in codes, all those posters that are all over the place taking up precedence.*’ (GP-27, metro). Translating and creating culturally appropriate posters was also raised in interviews, and as part of the Mackenzie’s Mission study, Arabic and Aboriginal and Torres Strait Islander posters were implemented.

## Discussion

We trialled two implementation strategies to support HCPs in offering carrier screening. Each approach had its strengths and specific idiosyncrasies. Designing implementation strategies to assist clinical practice behaviour change is challenging [19, 28]. A systematic approach during the initial design, execution, and evaluation phases that combines experience (study GCs) and behaviour change theory can inform long-term scaling-up of clinical interventions [29]. During the design phase, we drew on the TDF [30] to identify potential implementation strategies [31]. Here, the study GCs played a key role in the design phase with their contextual expertise, combining experiential knowledge with validated TDF constructs to develop robust and potentially feasible implementation strategies [14].

A key structural obstacle to the implementation strategies was time [32]. Many HCPs were enthusiastic to offer carrier screening as part of their general practice though not all were able to invest time up front to engage with the programme, thus affecting the viability of the implementation strategies [33]. Strategies that did not demand too many HCP resources were welcomed (e.g., waiting room posters) as preferable options regardless of changes in behaviour. Despite the implementation strategies being generally well received, those who trialled either the skills video or waiting room poster initiated less enrolments, although we note there was a reported increase in confidence and skills for the HCPS at these practices. It may be that once clinicians watched the video and employed the skills according to guidelines, offering testing took longer and so they stopped. This study took place against the backdrop of the COVID 19 pandemic and it is possible HCPs had competing demands. Proctor [34] posits feasibility of an intervention relies on convenience and circumstances. Despite employing a combined theory and experiential approach to codesign implementation strategies with frontline practitioners, we were unable to determine an appropriate level of convenience. However, this method does explain why a strategy did not work and provide the backdrop with which to identify strategies that would be workable in practice [35].

Educational videos and waiting room posters have previously been used effectively [36–38]. While these strategies impacted the TDF domains, suggesting perceived barriers to implementation were successfully targeted, little effect was seen on the number of couples accessing the study portal. This may be attributed to the barriers to implementation changing from the outset of the project (e.g., from technical skills) to later (e.g., belief about consequences). Longitudinal data collection would shed light on evolving barriers and so facilitate development of implementation strategies appropriate for different phases of HCP engagement with the project – from novice to expert.

Both the skills video and waiting room poster received mixed reviews from HCPs. Positives were being able to see the offering carrier screening behaviours modelled (skills video) and the simplicity of the waiting room poster. Criticisms included that the video was too long or had technical issues, and the poster was easily missed and lacked detail. Adaptation could potentially enhance the impact of the implementation strategies [39] either by the team designing the strategy or at a local level. For example, one local site designed an Indigenous Australian version of the waiting room poster to reflect their local population. Further adaptation could include a digital version to prompt couples to book a preconception appointment when visiting their HCP’s website. Central to adaptation is the identification of the core elements of the implementation strategy [40] that could be established through co-design with the study team and end users.

### Limitations

As to limitations, the execution phase coincided with the onset of the SARS-CoV-2 pandemic. Initially implementation strategies progressed, however, all activities were curtailed. The launch of the poster strategy was significantly delayed due to prolonged COVID-19 lockdowns and restrictions on waiting room use. Additionally, the study GCs will have impacted the execution phase, possibly introducing variation in the use of the implementation strategies and it is challenging to evaluate the contribution of the relationship-building their role required [39]. The TDF scores were not high, suggesting HCPs did not perceive many barriers to offering carrier screening. This could reflect the early adopter nature of the participants with HCPs not allocated using a randomised approach potentially introducing selection bias. To test TDF domains, we deployed a questionnaire that was previously validated with HCPs from acute hospitals in the UK [20], and Australia [41]. However, some domains did not demonstrate internal reliability in the context of carrier screening in primary care settings in Australia, limiting our analysis. The questionnaire was optional and had low completion rates; nevertheless, we could still apply learnings from the nine domains that demonstrated adequate reliability and the mixed methods approach ensured we captured a range of perspectives. Thus, our results offer an indication of how HCPs can be supported to offer carrier screening. Using a proxy measure for HCPs offering carrier screening presented as a challenge in capturing accurate measurement of behaviour change and may have led to an underestimation of true offer rates. Participants in our study will be early adopters and so more likely to have a favourable view of the implementation of a new practice [42]. Still, their feedback into the implementation strategies provided valuable insights to inform development of future support for HCPs offering carrier screening. Consumers were not part of the design team in this study. This was a pragmatic decision as consumers were actively being recruited into associated studies (e.g., perceptions of the Mackenzie’s Mission website and the experience of screening). Future work would benefit from the consumer input.

## Conclusion

HCPs play a central role in offering carrier screening to couples, and many will require support to incorporate this practice into already busy workloads [32]. The increased recognition of the need to go beyond efficacy when evaluating the implementation of complex implementation strategies demands consideration of a wide suite of tools [11]. We drew on experiential knowledge and behaviour change theory to design and trial two implementation strategies, based on reported barriers to offering carrier screening – skills and environmental context and resources. Both feasibility and evolving barriers presented as challenges to designing real world implementation strategies and although the content of the implementation strategies was indicated to reduce barriers, context (e.g., SARS-CoV-2 pandemic) played a key role [43], influencing the success of some implementation strategies. Finally, the need for adaptation was apparent to ensure implementation strategies were well suited to each local context.

### Supplementary Information

**Additional file 1. **Genetic Counsellor workshop. PowerPoint slides to guide discussion about using theory to inform implementation strategies.

**Additional file 2. **Structured debrief questions. Series of questions designed to monitor the strategy and guide the fortnightly debriefs with the study genetic counsellors.

**Additional file 3. **Internal reliability results of TDF informed questionnaire. Results table of the internal reliability analysis of the TDF domains.

**Additional file 4. **Skills video and poster strategy pre and post implementation cohort questionnaire TDF scores, mean (SD) range. Pre- and post-implementation questionnaire results (mean, SD and range) for the skills video and poster implementation strategy.

## Data Availability

The dataset(s) supporting the conclusions of this article is(are) included within the article (and its additional file(s)).

## Abbreviations

TDF: Theoretical Domains Framework
BCT: Behaviour Change Techniques
GC: Genetic Counsellor
HCP: Health Care Professional
TDF: Theoretical Domains Framework
RACGP: Royal Australian College of General Practitioners
RANZCOG: Royal Australian and New Zealand College of Obstetricians and Gynaecologists; RACGP, Royal Australian College of General Practitioners
