# Supplementary material for: Using a theory informed approach to design, execute, and evaluate implementation strategies to support offering reproductive genetic carrier screening in Australia
Source: BMC Health Serv Res. 2023 Nov 20;23:1276. doi: 10.1186/s12913-023-10053-1 (PMC10658900; doi:10.1186/s12913-023-10053-1)
Supplement: Supplementary file 1 — Additional file 1. Genetic Counsellor workshop. PowerPoint slides to guide discussion about using theory to inform implementation strategies. [file 12913_2023_10053_MOESM1_ESM.pptx]

## Slide 1
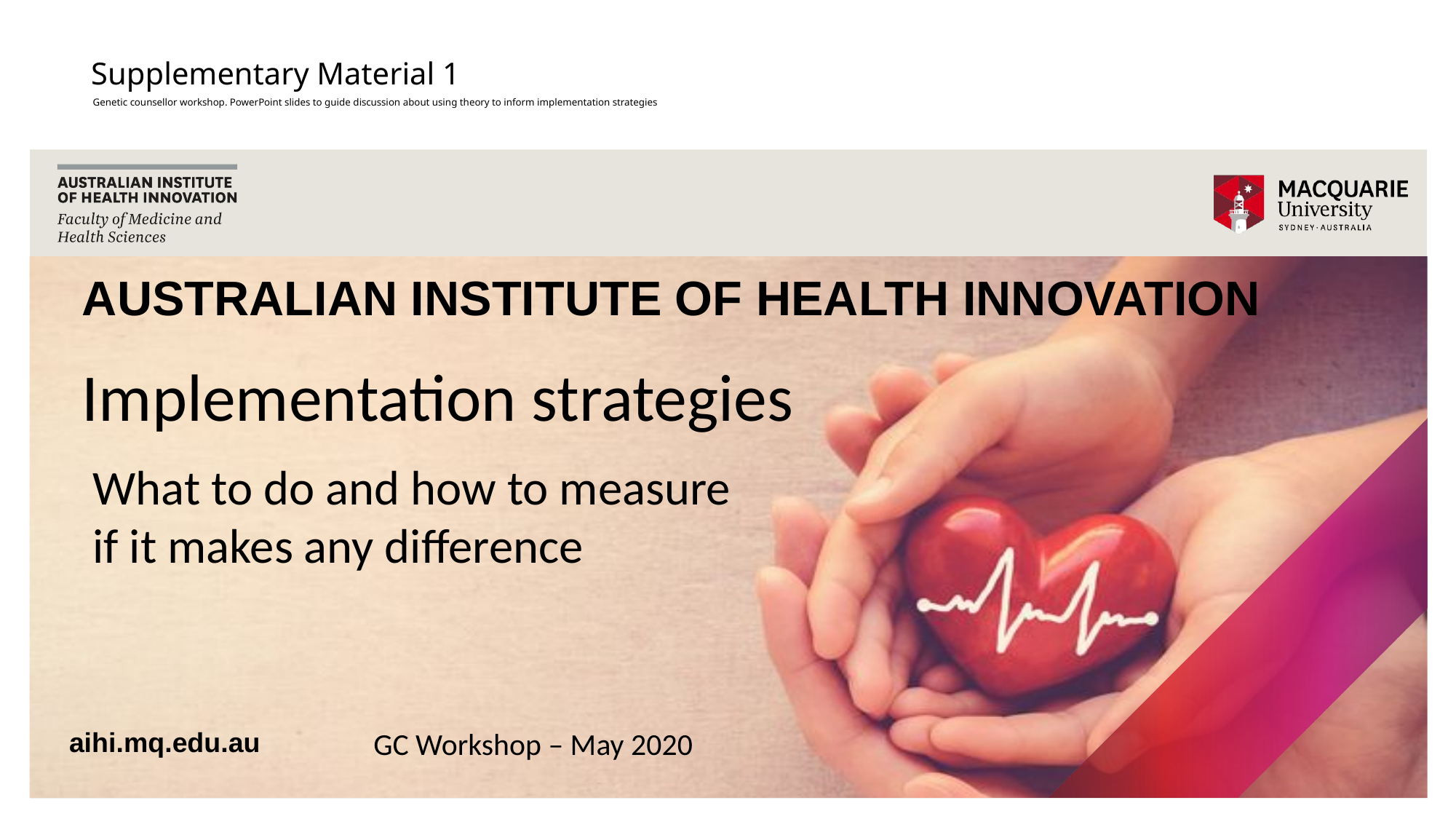

Supplementary Material 1
Genetic counsellor workshop. PowerPoint slides to guide discussion about using theory to inform implementation strategies
AUSTRALIAN INSTITUTE OF HEALTH INNOVATION
Implementation strategies
What to do and how to measure if it makes any difference
GC Workshop – May 2020
aihi.mq.edu.au

## Slide 2
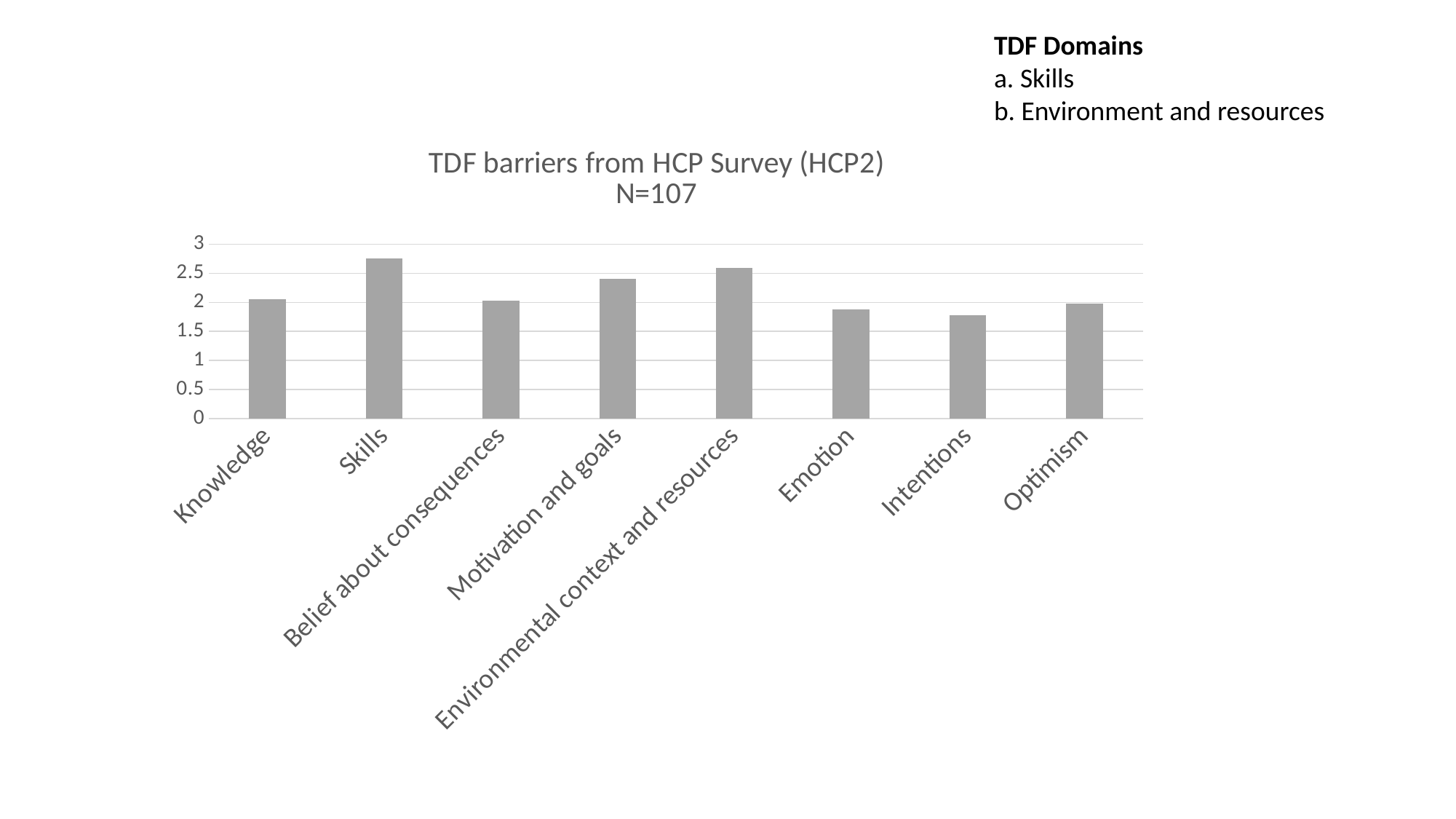

TDF Domains
a. Skills
b. Environment and resources
### Chart: TDF barriers from HCP Survey (HCP2)
N=107
| Category | |
|---|---|
| Knowledge | 2.049 |
| Skills | 2.76 |
| Belief about consequences | 2.034 |
| Motivation and goals | 2.399 |
| Environmental context and resources | 2.592 |
| Emotion | 1.883 |
| Intentions | 1.784 |
| Optimism | 1.974 |

## Slide 3
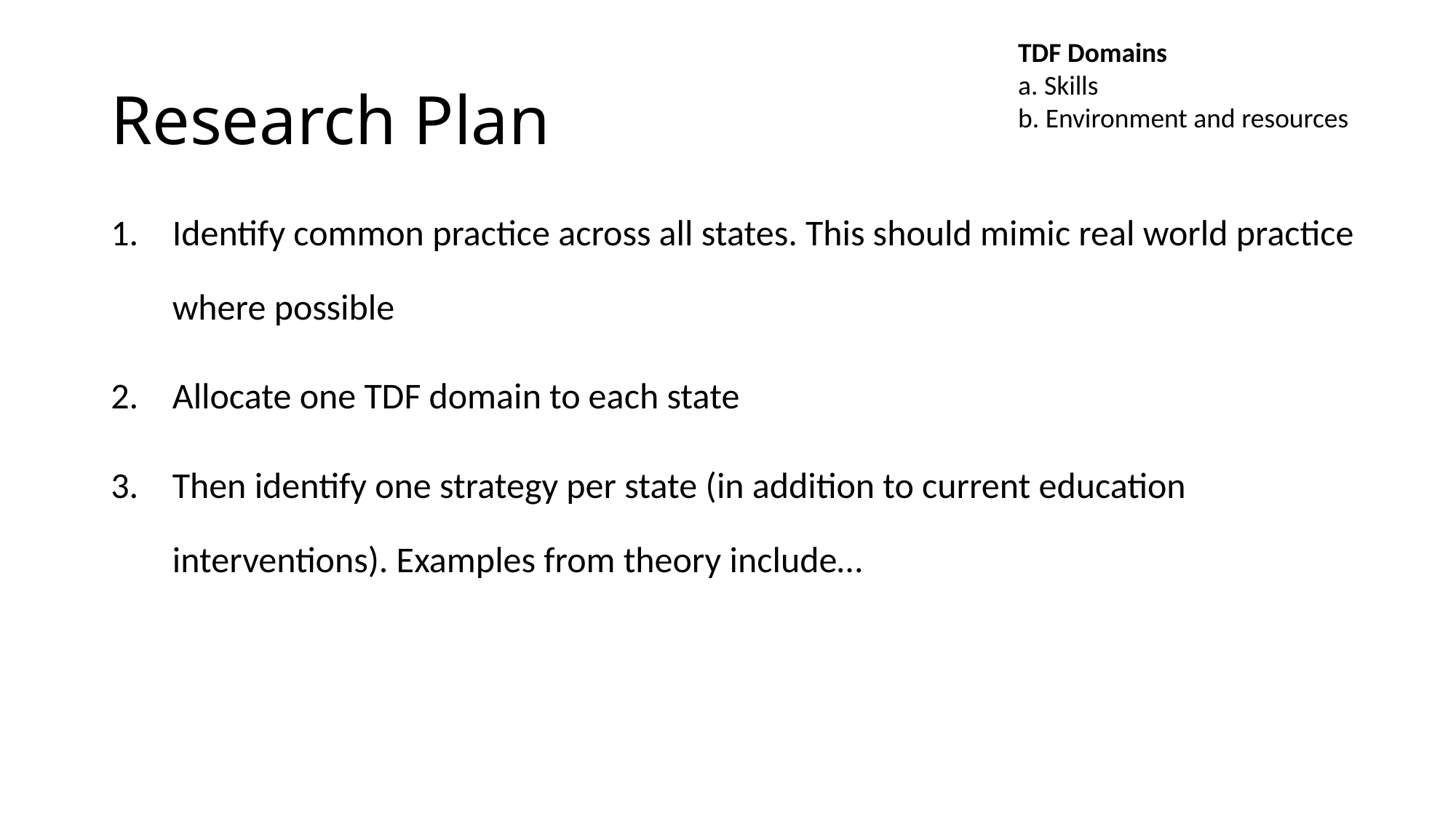

TDF Domains
a. Skills
b. Environment and resources
# Research Plan
Identify common practice across all states. This should mimic real world practice where possible
Allocate one TDF domain to each state
Then identify one strategy per state (in addition to current education interventions). Examples from theory include…

## Slide 4
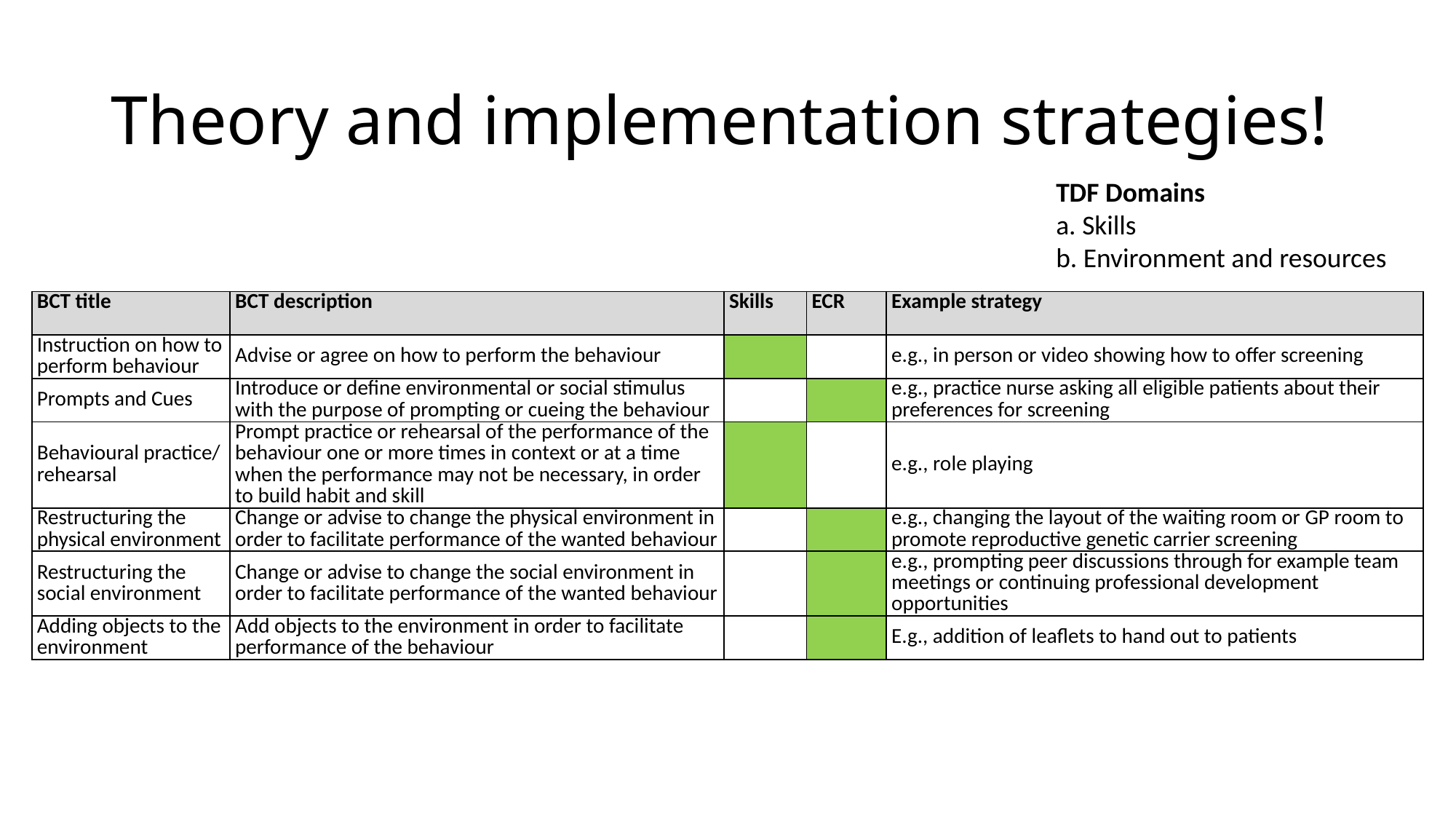

# Theory and implementation strategies!
TDF Domains
a. Skills
b. Environment and resources
| BCT title | BCT description | Skills | ECR | Example strategy |
| --- | --- | --- | --- | --- |
| Instruction on how to perform behaviour | Advise or agree on how to perform the behaviour | | | e.g., in person or video showing how to offer screening |
| Prompts and Cues | Introduce or define environmental or social stimulus with the purpose of prompting or cueing the behaviour | | | e.g., practice nurse asking all eligible patients about their preferences for screening |
| Behavioural practice/ rehearsal | Prompt practice or rehearsal of the performance of the behaviour one or more times in context or at a time when the performance may not be necessary, in order to build habit and skill | | | e.g., role playing |
| Restructuring the physical environment | Change or advise to change the physical environment in order to facilitate performance of the wanted behaviour | | | e.g., changing the layout of the waiting room or GP room to promote reproductive genetic carrier screening |
| Restructuring the social environment | Change or advise to change the social environment in order to facilitate performance of the wanted behaviour | | | e.g., prompting peer discussions through for example team meetings or continuing professional development opportunities |
| Adding objects to the environment | Add objects to the environment in order to facilitate performance of the behaviour | | | E.g., addition of leaflets to hand out to patients |

## Slide 5
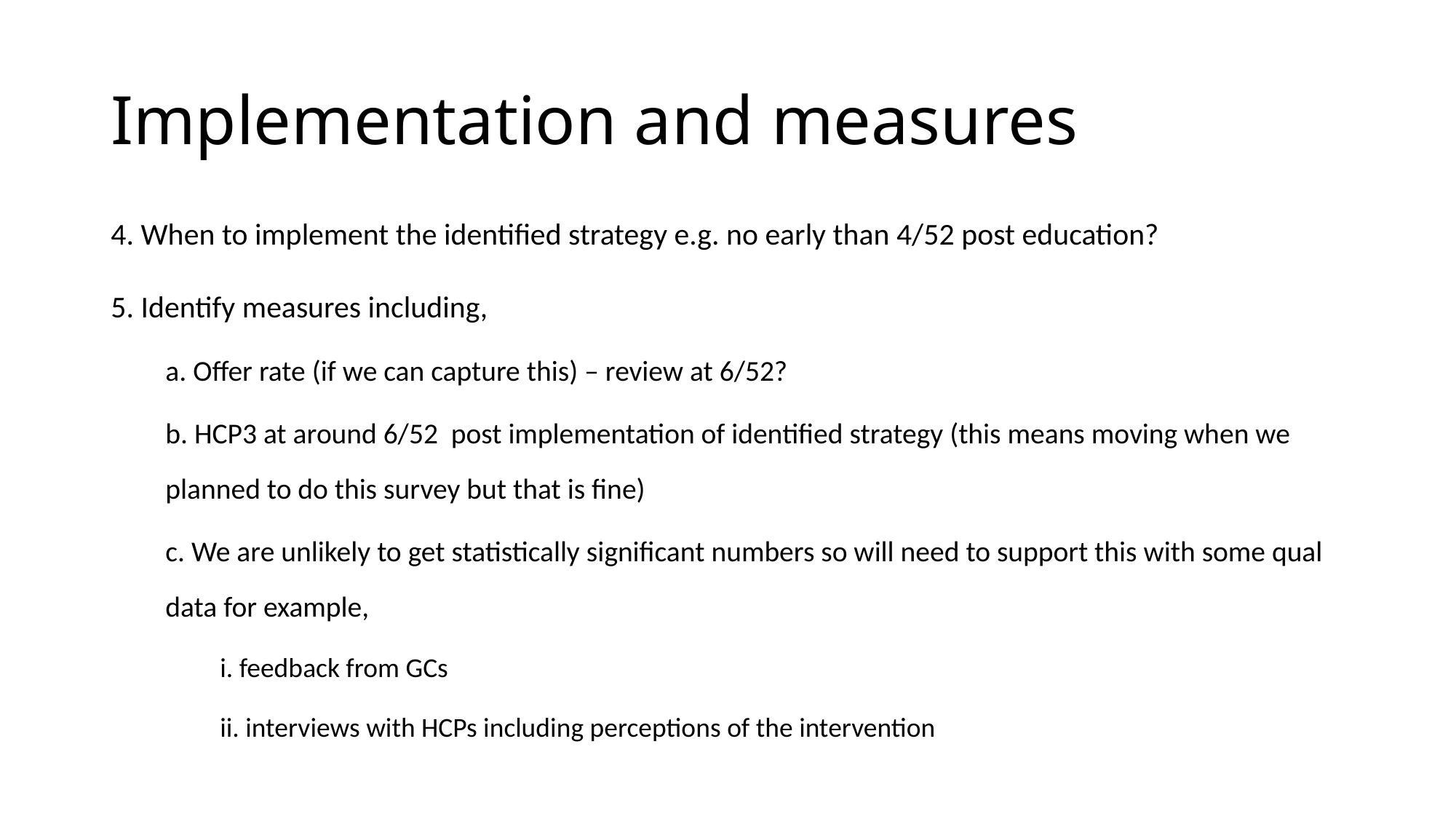

# Implementation and measures
4. When to implement the identified strategy e.g. no early than 4/52 post education?
5. Identify measures including,
a. Offer rate (if we can capture this) – review at 6/52?
b. HCP3 at around 6/52 post implementation of identified strategy (this means moving when we planned to do this survey but that is fine)
c. We are unlikely to get statistically significant numbers so will need to support this with some qual data for example,
i. feedback from GCs
ii. interviews with HCPs including perceptions of the intervention

## Slide 6
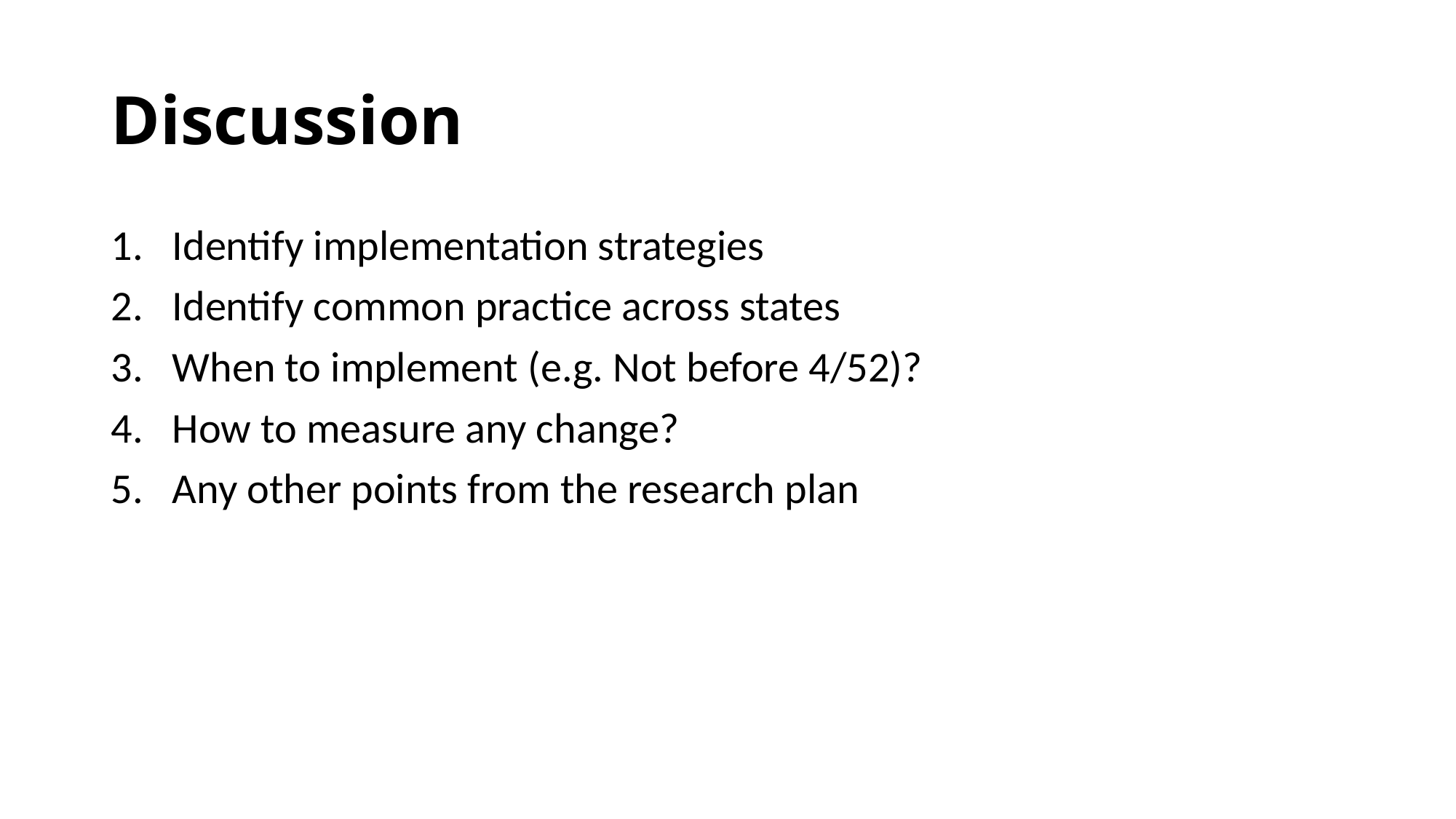

# Discussion
Identify implementation strategies
Identify common practice across states
When to implement (e.g. Not before 4/52)?
How to measure any change?
Any other points from the research plan
