# Supplementary material for: Using a theory informed approach to design, execute, and evaluate implementation strategies to support offering reproductive genetic carrier screening in Australia
Source: BMC Health Serv Res. 2023 Nov 20;23:1276. doi: 10.1186/s12913-023-10053-1 (PMC10658900; doi:10.1186/s12913-023-10053-1)
Supplement: Supplementary file 2 — Additional file 2. Structured debrief questions. Series of questions designed to monitor the strategy and guide the fortnightly debriefs with the study genetic counsellors. [file 12913_2023_10053_MOESM2_ESM.docx]

Supplementary Material 2: Structured debrief questions. Series of questions designed to monitor the strategy and guide the fortnightly debriefs with the study genetic counsellors

| Structured Genetic Counsellor debriefs |
| --- |
| How's it going? |
| Have there been any comments from health care professionals about the intervention? |
| Any barriers to using the intervention? |
| Any enablers to using the intervention? |
| Have you seen any changes in contact from health care professionals/ offer/take up rates? |
| Themes from phone calls with health care professionals? |
| Any thoughts on how to change the intervention? |
| Are health care professionals providing the correct information? |
| Any other thoughts/comments |
