# Supplementary material for: Using a theory informed approach to design, execute, and evaluate implementation strategies to support offering reproductive genetic carrier screening in Australia
Source: BMC Health Serv Res. 2023 Nov 20;23:1276. doi: 10.1186/s12913-023-10053-1 (PMC10658900; doi:10.1186/s12913-023-10053-1)
Supplement: Supplementary file 3 — Additional file 3. Internal reliability results of TDF informed questionnaire. Results table of the internal reliability analysis of the TDF domains. [file 12913_2023_10053_MOESM3_ESM.docx]

Supplementary Material 3: Internal reliability results of TDF informed questionnaire. Results table of the internal reliability analysis of the TDF domains

| TDF Domain | Spearman-Brown  (2 items) | Cronbach’s alpha  (3 items) |
| --- | --- | --- |
| Knowledge | 0.636 | n/a |
| Skills | 0.725 | n/a |
| Social/professional role & identity | 0.368* | n/a |
| Beliefs about capabilities | 0.472* | n/a |
| Beliefs about consequences | 0.522 | n/a |
| Motivation & goals | 0.501 | n/a |
| Memory, attention & decision processes | 0.281* | n/a |
| Environmental context & resources | n/a | 0.707 |
| Social influences | 0.183* | n/a |
| Emotion | 0.862 | n/a |
| Behaviour regulation & action planning | 0.563 | n/a |
| Goals | n/a | 0.487* |
| Intentions | n/a | 0.715 |
| Reinforcement | n/a | 0.373* |
| Optimism | n/a | 0.813 |

Note: *denotes domain did not demonstrate adequate internal reliability
