# Supplementary material for: Using a theory informed approach to design, execute, and evaluate implementation strategies to support offering reproductive genetic carrier screening in Australia
Source: BMC Health Serv Res. 2023 Nov 20;23:1276. doi: 10.1186/s12913-023-10053-1 (PMC10658900; doi:10.1186/s12913-023-10053-1)
Supplement: Supplementary file 4 — Additional file 4. Skills video and poster strategy pre and post implementation cohort questionnaire TDF scores, mean (SD) range. Pre- and post-implementation questionnaire results (mean, SD and range) for the skills video and poster implementation strategy. [file 12913_2023_10053_MOESM4_ESM.docx]

Supplementary Material 4: Skills video and poster strategy pre and post implementation cohort questionnaire TDF scores, mean (SD) range. Pre- and post-implementation questionnaire results (mean, SD and range) for the skills video and poster implementation strategy.

|  | Skills video | | | No skills video | | |
| --- | --- | --- | --- | --- | --- | --- |
|  | Pre-implementation  (n= 11) | Post-implementation^1^  (n= 15) | Difference | Pre-implementation  (n= 10) | Post-implementation^1^  (n= 17) | Difference |
| TDF domain |  |  |  |  |  |  |
| Knowledge | 2.59 (0.70) 2-4 | 1.90 (0.47) 1-2.5 | -0.69 | 2.40 (0.61) 1.5-3 | 1.94 (0.34) 1.5-2.5 | -0.46 |
| Skills | 3.50 (1.09) 2-5 | 2.60 (0.94) 1.5-5 | -0.90 | 3.05 (1.01) 2-4^2^ | 2.64 (0.87) 1.5-5 | -0.41 |
| Behavioural regulation & action planning | 2.27 (0.46) 2-3.5 | 2.06 (0.59) 1-3.5 | -0.21 | 2.05 (0.64) 1-3 | 1.97 (0.62) 1-3.5 | -0.08 |
| Environmental context & resources | 3.24 (0.63) 2.33-4 | 2.60 (0.61) 2-4 | -0.64 | 2.86 (0.50) 2-3.66 | 2.74 (0.62) 1.66-3.66 | -0.12 |
| Beliefs about consequences | 2.18 (0.33) 1.5-2.5 | 2.26 (0.41) 1.5-3 | +0.08 | 2.30 (0.67) 1.5-4 | 2.29 (0.58) 1-3.5 | -0.01 |
| Motivation & Goals | 2.72 (0.46) 2-3.5 | 2.66 (0.74) 1.5-4 | -0.06 | 2.25 (0.54) 1.5-3 | 2.73 (0.86) 1.5-5 | +0.48 |
| Emotion | 2.40 (0.76) 2-4.5 | 1.83 (0.24) 1.5-2 | -0.57 | 2.30 (0.58) 1-3 | 2.00 (0.81) 1-3.5 | -0.30 |
|  | Poster | | | No poster | | |
|  | Pre-implementation  (n= 36) | Post-implementation^1^  (n= 26) | Difference | Pre- implementation  (n= 17) | Post-implementation^1^  (n= 14) | Difference |
| TDF domain |  |  |  |  |  |  |
| Knowledge | 2.13 (0.75) 1-5 | 1.75 (0.66) 1-3.5 | -0.38 | 2.20 (0.88) 1-4 | 1.82 (0.77) 1-3.5 | -0.38 |
| Skills | 3.02 (1.05) 1-5^2^ | 2.48 (0.97) 1-4.5 | -0.54 | 3.58 (0.59) 3-4.5 | 2.67 (0.86) 1-4 | -0.91 |
| Behavioural regulation & action planning | 2.14 (0.65) 1-3.5^2^ | 1.92 (0.57) 1-3 | -0.22 | 2.50 (0.75) 1-4 | 2.42 (0.82) 1-4 | -0.08 |
| Environmental context & resources | 2.82 (0.77) 1-4.33^3^ | 2.26 (0.72) 1-4 | -0.56 | 2.98 (0.67) 2-4 | 2.78 (0.60) 2-4 | -0.20 |
| Beliefs about consequences | 2.26 (0.49) 1-3.5 | 2.13 (0.59) 1-3.5 | -0.13 | 2.29 (0.61) 1-3 | 2.53 (1.02) 1-4 | +0.24 |
| Motivation & Goals | 2.56 (0.70) 1-3.5 | 2.48 (0.79) 1-4 | -0.08 | 2.67 (0.93) 1-4 | 2.89 (0.56) 2-3.5 | +0.22 |
| Emotion | 2.25 (0.90) 1-4^2^ | 1.78 (0.68) 1-3 | -0.47 | 2.41 (1.14) 1-4 | 2.03 (0.92) 1-4 | -0.38 |

Note: Analysis excludes missing cases; ^1^ 8 weeks after implementation strategy; ^2^ Missing data for 1 participant; ^3^ Missing data for 2 participants
